# Disease resistance features of the executor *R* gene *Xa7* reveal novel insights into the interaction between rice and *Xanthomonas oryzae* pv. *oryzae*

**DOI:** 10.3389/fpls.2024.1365989

**Published:** 2024-04-03

**Authors:** Lumei He, Pengcheng Liu, Le Mei, Huichao Luo, Tingxuan Ban, Xifeng Chen, Bojun Ma

**Affiliations:** College of Life Sciences, Zhejiang Normal University, Jinhua, China

**Keywords:** rice, bacterial blight, executor *R* genes, *Xa7*, rice-*Xoo* interaction

## Abstract

Bacterial blight (BB), caused by *Xanthomonas oryzae* pv. *oryzae* (*Xoo*), is a widespread and destructive disease in rice production. Previously, we cloned an executor *R* gene, *Xa7*, which confers durable and broad-spectrum resistance to BB. Here, we further confirmed that the transcription activator-like effector (TALE) AvrXa7 in *Xoo* strains could directly bind to the effector-binding element (EBE) in the promoter of the *Xa7* gene. Other executor *R* genes (*Xa7*, *Xa10*, *Xa23*, and *Xa27*) driven by the promoter of the *Xa7* gene could be activated by AvrXa7 and trigger the hypersensitive response (HR) in tobacco leaves. When the expression of the *Xa23* gene was driven by the *Xa7* promoter, the transgenic rice plants displayed a similar resistance spectrum as the *Xa7* gene, demonstrating that the disease resistance characteristics of executor *R* genes are mainly determined by their induction patterns. *Xa7* gene is induced locally by *Xoo* in the infected leaves, and its induction not only inhibited the growth of incompatible strains but also enhanced the resistance of rice plants to compatible strains, which overcame the shortcomings of its race-specific resistance. Transcriptome analysis of the *Xa7* gene constitutive expression in rice plants displayed that *Xa7*-mediated disease resistance was related to the biosynthesis of lignin and thus enhanced resistance to *Xoo*. Overall, our results provided novel insights and important resources for further clarifying the molecular mechanisms of the executor *R* genes.

## Introduction

*Xanthomonas oryzae* pv. *oryzae* (*Xoo*), the causal agent of bacterial blight (BB) disease in rice (*Oryza sativa* L.), is a notorious plant pathogenic bacteria that greatly affects global food security (Jiang et al., 2020). The interaction between *Xoo* strains and rice plants has been regarded as a classic model for studying bacteria–plant interaction (NIÑO-LIU et al., 2006). *Xoo* strains inject transcription activator-like effectors (TALEs) into rice plant cells through their type III secretion system (T3SS), which is capable of activating the transcription of specific genes. Among them are the susceptibility genes, such as the SWEETs (sugars will eventually be exported transporter) family members, benefiting their own reproduction and invasion, a phenomenon referred to as effector-triggered susceptibility (ETS) (Boch et al., 2014). To combat the attack of *Xoo*, plants evolved several types of resistance (*R*) genes that can trap *Xoo* TALEs and trigger immunity in host cells, which is known as effector-triggered immunity (ETI) (Gu et al., 2005). A typical TALE usually has a secretion signal motif in its N-terminal, a binding domain for target recognition, a transcription factor binding (TFB) domain, highly conserved nuclear localization signals (NLSs), and acidic transcription activator-like domains (AADs) (Szurek et al., 2001). Distinctively, the central repeat domain of TALEs contains a variable number of tandemly arranged 33 to 34 amino acid repeats, and the 12th and 13th, two residues in each repeat called repeat variable di-residues (RVDs), can recognize a specific nucleotide (Boch et al., 2009). Therefore, TALEs can specifically bind to the promoter of *R* or *S* genes by recognizing the effector-binding elements (EBEs) and then activating their expression (Schornack et al., 2006)

Executor *R* genes are regarded as a unique type of disease resistance genes in plants, which can specifically trap TALEs of plant pathogens and trigger strong hypersensitive response (HR) and directly programmed cell death (PCD) in the host. Up to now, six genes belonging to this type have been cloned; among them, *Bs3* (Römer et al., 2007) and *Bs4C* (Strauss et al., 2012) were isolated from pepper (*Capsicum frutescens*), while *Xa27* (Gu et al., 2005), *Xa10* (Tian et al., 2014), *Xa23* (Wang et al., 2015), and *Xa7* (Chen et al., 2021; Luo et al., 2021; Wang et al., 2021) were cloned from rice. Interestingly, each identified executor *R* gene (except *Xa10*) has an identical coding sequence (CDS) with its susceptible allele but only differs in the promoter, which has an EBE sequence specifically recognized by a cognate TALE of pathogens, such as AvrBs3, AvrBs4, AvrXa27, AvrXa10, AvrXa23, or AvrXa7 (Ballvora et al., 2001; Szurek et al., 2002; Ji et al., 2022b). Therefore, the executor *R* genes are usually “silent” and only activated to trigger rapid host-cell death when the invading pathogens are present, effectively preventing further pathogen attacks (Nowack et al., 2022). Evidence has shown that the expression of the executor *R* genes could trigger cell death in both plant and animal cells (Strauss et al., 2012; Tian et al., 2014; Chen et al., 2021), suggesting that the defense pathway of these executor *R* genes may be conserved. However, only the executor *R* gene *Bs3* has been known to encode a putative flavin-containing monooxygenase (FMO), which may be involved in the metabolism of reactive oxygen species (Krönauer et al., 2019). Unlike the *Bs3* gene, the other five executor *R* genes encode small unknown proteins, which have 113 to 164 amino acids with two to four transmembrane domains (Ji et al., 2022b; Nowack et al., 2022). In addition, those executor *R* genes have low similarities in nucleotide sequence and no homology with other R proteins that have been identified. To date, little is known about the molecular mechanisms of the executor *R* genes.

Recently, we cloned the rice executor *R* gene *Xa7* (Chen et al., 2021; Luo et al., 2021; Wang et al., 2021), which has been confirmed to be a broad-spectrum and durable BB resistance gene (Vera Cruz et al., 2000; White and Yang, 2009). We found that the expression of *Xa7* was strongly induced by the incompatible *Xoo* strains, and a putative EBE sequence (named EBE_AvrXa7_) in the promoter of *Xa7* could be perfectly matched by the RVDs of AvrXa7 (Chen et al., 2021). AvrXa7 has been reported to play dual roles as both virulence factor and avirulence effector in *Xoo*, which makes the *Xa7* gene a broad-spectrum resistance against BB disease (Zhang et al., 2015). To study the disease resistance mechanism of *Xa7* gene, here, we confirmed the interaction between AvrXa7 and the EBE_AvrXa7_, constructed the transgenic plants with different executor *R* genes driven by the *Xa7* promoter to test BB resistance, analyzed the relationship between the expression pattern of *Xa7* gene and the infection of *Xoo*, and explored the defense response pathway of *Xa7* gene by RNA-seq. Our results will be helpful and provide novel insights for future research on the molecular mechanisms of the executor *R* genes.

## Materials and methods

### Plant materials and growth conditions

IR24 is a susceptible *indica* rice variety without the *Xa7* gene, while IRBB7 is a near-isogenic line of IR24 containing the *Xa7* gene. A *japonica* rice variety Zhonghua11 (ZH11) does not contain the *Xa7* gene locus was selected as the wild-type control and transgenic recipient. Transgenic lines constitutively expressing the *Xa7* gene (*Xa7-OE-1* and *Xa7-OE-2*) were obtained in our previous research (Chen et al., 2021). The detailed information on rice varieties used in this study is listed in **Supplementary Table S3**. All rice plants were grown in natural paddy fields during summer in southern China. Tobacco (*Nicotiana benthamiana*) plants were cultured in a growth chamber at 25°C (16-hour light/8-hour dark).

### Yeast one-hybrid assay

A synthetic DNA fragment containing three tandem repeats of the predicted EBE_AvrXa7_ sequence was inserted into the p*LacZ* vector (digested with *Nco*I) *via* homologous recombination. Subsequently, the engineered p*LacZ* construct was linearized with *Nco*I and used for the transformation of the yeast strain YM4271. Positive colonies were able to grow on a nutrient-deficient medium (SD/-Ura) (Breton et al., 2016). A vector expressing the fusion protein of the GAL4 activation domain (AD) and the RVDs of AvrXa7 was constructed using the pGADT7 vector and then transformed into YM4271 harboring the corresponding p*LacZ* vector. Positive colonies were screened on a nutrient-deficient plate (SD/-Ura/-Leu). β-Galactosidase activity was assessed using a commercially available luminescent β-galactosidase substrate Beta-Glo, which was cleaved and then released d-luciferin for detection using firefly luciferase.

### HR assay in tobacco leaves

Constructs of *35S::avrXa7*, *Xa7_pro_::Xa7*, *Xa7_pro_::Xa10*, *Xa7_pro_::Xa23*, and *Xa7_pro_::Xa27* were transformed into *Agrobacterium tumefaciens* strain EHA105 using the heat-shock method. The bacteria were cultured in 20 mL of Lysogeny Broth (LB) liquid medium with 50 mg/L kanamycin and 25 mg/L rifampin until the density reached OD_600 = _0.6. The qualified bacterial cells were then collected by centrifugation at 4°C for 10 min at 3,000 rpm and re-suspended in an inoculation medium containing 10 mM MgCl_2_, 10 mM MES, and 200 μM AS. Leaves of 4-week-old tobacco were inoculated using needleless syringes following the method described by Kay et al. (2007). The inoculated leaves were harvested after 2 days of inoculation and stained using 3,3′-diaminobenzidine (DAB), as described by Daudi and O'Brien (2012).

### Vector construction and genetic transformation

The PrimeSTAR HS DNA Polymerase Kit (TaKaRa, Mountain View, CA, USA) was used to amplify the fragments of interest with specific primer pairs listed in **Supplementary Table S4**. The PCR products were then inserted into the frame vector pCAMBIA1300 using an In-Fusion HD Cloning Kit (TaKaRa). The verified vectors were transformed into the rice *japonica* variety Zhonghua11 using the *Agrobacterium*-mediated method, as described previously (Nishimura et al., 2006). DNA sequences of used constructs are shown in **Supplementary 5**.

### Inoculation of *Xoo* strains

The *Xoo* strains PXO86 and PXO99 were obtained from the International Rice Research Institute (IRRI) in the Philippines. The engineered strain PXO99*^avrXa7^* was created by our group in the previous research, which contained an *avrXa7* overexpression construct in the PXO99 strain (Chen et al., 2021). Among them, PXO86 and PXO99*^avrXa7^* are incompatible strains of the *Xa7* gene in the rice variety IRBB7, while PXO99 is a compatible strain of the *Xa7* gene in IRBB7. The strains were initially cultured on agar medium containing 20 g/L sucrose, 5 g/L peptone, 0.5 g/L Ca(NO_3_)_2_, 0.43 g/L Na_2_HPO_4_, and 0.05 g/L FeSO_4_ at 28°C for 2 days. The bacterial colonies were then eluted with sterile water and diluted to a concentration of OD_600 = _1.0 for subsequent inoculation. The leaf-tip clipping method (Kauffman et al., 1973) was used for inoculation of the *Xoo* strains, and lesions on the inoculated leaves were measured 2 weeks after inoculation to evaluate BB resistance (Yin et al., 2000).

### GUS-staining assay

During the amplification of the *Xa7* promoter, we obtained a mutated *Xa7* promoter, which has two cytosine bases deleted; as a result, the *Xa7* gene driven by the mutated *Xa7* promoter could not induced by AvrXa7. Thus, we chose this promoter as a native control. We inoculated the leaves of *Xa7_pro_::Gus* and *Xa7_mpro_::Gus* transgenic plants with PXO86 at the early stage of tillering, and we detected Gus activity by submerging the infected leaves in GUS-staining solution (Zhang et al., 2018).

### RT-qPCR assay

Total RNA was extracted from rice leaves using the RNeasy Plant Mini Kit (Qiagen, Valencia, CA, USA) and was purified with the RNase-Free DNase Set (Qiagen) in accordance with the manufacturer’s instructions. Synthesis of first-strand cDNAs from the extracted RNA was performed using the M-MLV Reverse Transcriptase Kit (Promega, Madison, WI, USA) according to the manufacturer’s instructions. Real-time PCR (qRT-PCR) was carried out using the Fast SYBR Green Master Mix Reagent (Applied Biosystems, Foster City, CA, USA) on an ABI7500 Real-time PCR System. For qRT-PCR in rice, the housekeeping gene *Actin* was used as the internal control, while in *N. benthamiana*, the expression of the *NbGAPDH* was used as the internal control. The expression of target genes was quantified using the 2^−ΔΔCt^ method (Livak and Schmittgen, 2001). Standard errors were calculated based on a minimum of three biological replicates. The primers used are listed in **Supplementary Table S4**.

### Compatible and incompatible strain treatment assay

The leaves of IRBB7 were inoculated with the positive control strain PXO86 (incompatible), the negative control strain PXO99 (compatible), and a mixed control strain (PXO86+PXO99) at the same time, while the remaining treatments were first inoculated with PXO86 strain simultaneously to induce the expression of the *Xa7* gene and then inoculated with PXO99 strain at 1 day, 2 days, and 3 days after the initial inoculation, separately. One-month-old rice plants after transplantation were inoculated with *Xoo* strains at approximately 1 × 10^9^ cfu/mL using the leaf-clipping method described above.

### RNA-seq analysis

Leaves of the transgenic plants of *Xa7-OE-1* and *Xa7-OE-2*, as well as the wild-type control ZH11, were harvested with three biological replicates at the tillering stage (2 months old) and immediately frozen in liquid nitrogen. All samples were sent to the Beijing Genomics Institute (BGI) for RNA-seq using a HiSeq 2500 sequencing system (Illumina, San Diego, CA, USA). The raw data were processed to remove the adaptor sequences and low-quality reads by SOAPnuke (Kawahara et al., 2013). Then, the cleaning reads were mapped onto the reference genome sequence of Nipponbare (http://rice.plantbiology.msu.edu/) by Bowtie2 (Cock et al., 2010; Langmead and Salzberg, 2012). The transcriptional level of expression genes was calculated using RSEM (Li and Dewey, 2011). Differentially expressed genes (DEGs) were identified using DEGseq (Wang et al., 2010) with the Log_2_FC ≥1 or ≤−1 and significance adjusted at *p* ≤ 0.05. Enrichment analysis of Kyoto Encyclopedia of Genes and Genomes (KEGG) pathways of the DEGs was performed on the BGI website (https://report.bgi.com). The raw sequencing data were deposited in the National Center for Biotechnology Information (NCBI) (https://www.ncbi.nlm.nih.gov/) with the BioProject accession number PRJNA954312.

### Determination of lignin content

The leaves of ZH11, *Xa7-OE-1*, and *Xa7-OE-2* at the early stage of tillering were sampled and then ground into powder after fully drying for 96 hours. The powder was filtered using a 40 mesh sieve, and then 5 mg was weighed for the determination of lignin content. The measurement method was carried out according to the handbook of the Lignin Content Assay Kit (JC2203-S, China). The samples were mixed with acetic acid, and absorbance was measured at 280 nm. The content of lignin (%) was calculated using a computed formula with an extinction coefficient of 17.75 mL·mg^−1^·cm^−1^ for rice.

### Statistical methods and significance analysis

Data plotting and statistical analysis were performed using GraphPad Prism 8.0 software (https://www.graphpad.com/). Data are shown as the means ± SD, and significance analysis was conducted using a two-tailed Student’s *t*-test or a one-way analysis of variance (ANOVA) with least significant difference (LSD) multiple comparisons test. Asterisks represent statistical significance as *p < 0.05 and **p < 0.01.

## Results

### AvrXa7 directly binds to the EBE_AvrXa7_ in the promoter of the *Xa7* gene

**Figure 1:**
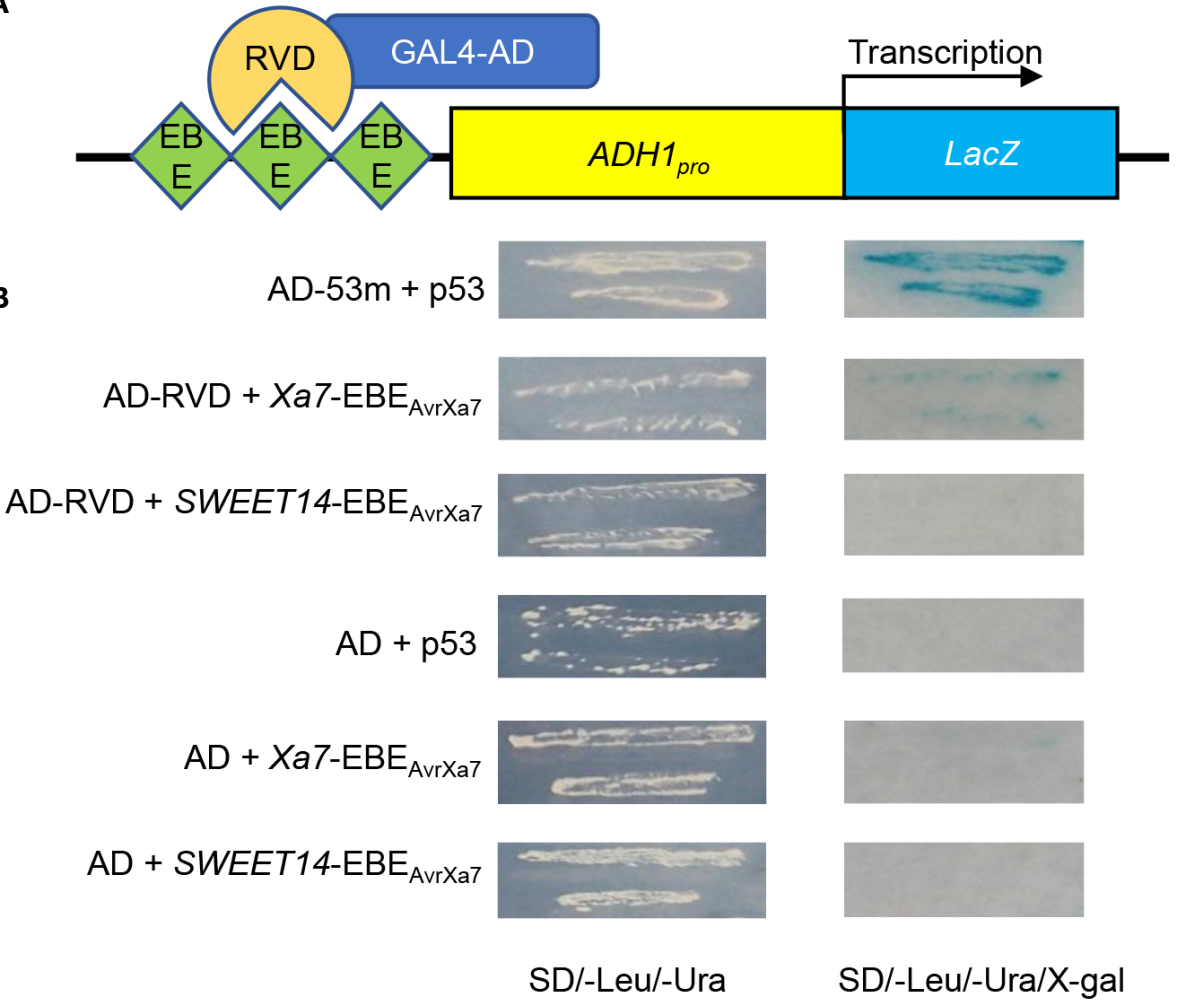
Identification of the interaction between AvrXa7 and *Xa7*-EBE_AvrXa7_. **(A)** Schematic diagram of the yeast one-hybrid system used in this study. Three tandem repeats of *Xa7*-EBE_AvrXa7_ and *SWEET14*-EBE_AvrXa7_ were inserted upstream of the *LacZ* gene. The repeat variable di-residues (RVDs) of AvrXA7 (AD-RVD) were fused with the activation domain (AD) of GAL4. *ADH1_pro_* refers to the constitutive *ADH1* promoter, which drives the expression of the *LacZ* gene. **(B)** The β-gal activity test results of different transformants. The interaction between p53Blue and AD-53m was used as the positive control. p*LacZ*-EBE_AvrXa7_ represents the reporter vector, while AD-RVD represents the effector vector.

In a previous study, a 26-bp putative EBE_AvrXa7_ sequence (ATAACCCCCCCCCCCCCAGATAACCA) was predicted in the promoter of the *Xa7* gene, which matched perfectly with the 25.5 RVDs of AvrXa7 based on the recognition principle between TALEs and EBEs (Chen et al., 2021). To confirm that AvrXa7 can indeed interact with EBE_AvrXa7_, a yeast one-hybrid assay was carried out. In the reporter vector, three tandem repeats of both *Xa7*-EBE_AvrXa7_ and *SWEET14*-EBE_AvrXa7_ were inserted upstream of the *LacZ* gene, while the effector vector (AD-RVD) expressed the RVDs of AvrXa7 fused with the GAL4 activation domain (**Figure 1A**). The yeast strains co-transformed with *Xa7*-EBE_AvrXa7_ and AD-RVD grew on a nutrient-deficient medium and showed significant β-gal activity, as detected by blue staining (**Figure 1B**). These results indicate that AvrXa7 can directly bind to the EBE_AvrXa7_ sequence in the promoter of the *Xa7* gene.

### AvrXa7 activates the executor *R* genes driven by the *Xa7* promoter in tobacco

**Figure 2:**
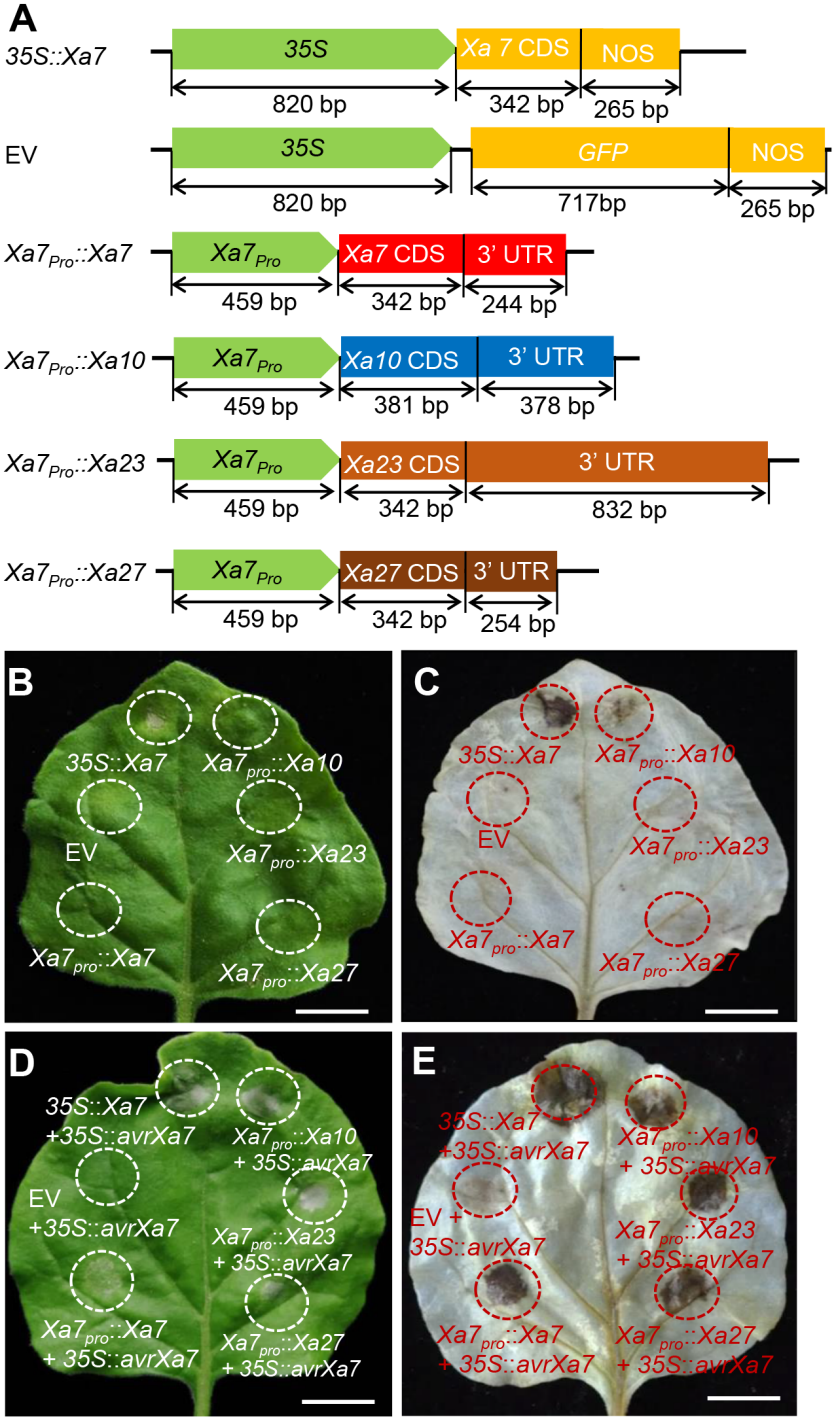
Executor *R* genes activated by the *Xa7* promoter triggered hypersensitive response (HR) in tobacco leaves. **(A)** Vector diagrams of positive control *35S::Xa7*, negative control empty vector (EV), and four executor *R* genes driven by the *Xa7* promoter, named *Xa7_pro_::Xa7*, *Xa7_pro_::Xa10*, *Xa7_pro_::Xa23*, and *Xa7_pro_::Xa27*. **(B)** HR assays in tobacco leaves. *Agrobacterium tumefaciens* containing different vectors were injected into tobacco leaves using needleless syringes. *35S::Xa7* was selected as the positive control, while EV as the negative control. The infiltrated areas are outlined with dashed circles. **(C)** The results of 3,3′-diaminobenzidine (DAB) staining assay. The corresponding leaf from **(B)** was stained with DAB. **(D)** Phenotype of vectors co-injected with the *35S::AvrXa7* in tobacco leaves. **(E)** The DAB staining result of corresponding leaves in **(D)**.

To investigate whether the promoter of the *Xa7* gene can activate other executor *R* genes by binding with AvrXa7, six constructs were created (**Figure 2A**) and transiently transferred into tobacco for HR analysis—a plant-specific basic immunity that causes infected cell death *via* the bursts of reactive oxygen species (ROS; Lorrain et al., 2003). Among them, four constructs containing the executor *R* gene *Xa7*, *Xa10*, *Xa23*, and *Xa27* were driven by the native promoter of *Xa7* (*Xa7_pro_*). The remaining two constructs were driven by the *CaMV35S* promoter that constitutively expressed *Xa7* or *GFP* (**Figure 2A**). The empty vector (EV) was used as a negative control. The results showed that significant cell death and excessive ROS accumulation occurred only when transforming the *35S::Xa7* or co-transforming the *Xa7_pro_::executor* (*Xa7*, *Xa10*, *Xa23*, or *Xa27*) with *35S::avrXa7* in the tobacco leaves. No or only slight (*Xa7_pro_::Xa10*) HR phenotype was observed in other situations (**Figures 2B–E**; **Supplementary Figure S1**). The above results suggested that the promoter of the *Xa7* gene can activate other executor *R* genes to trigger HR in plants by inoculation with AvrXa7.

### The *Xa7* gene promoter can endow *Xa23* with similar disease resistance characteristics

**Figure 3:**
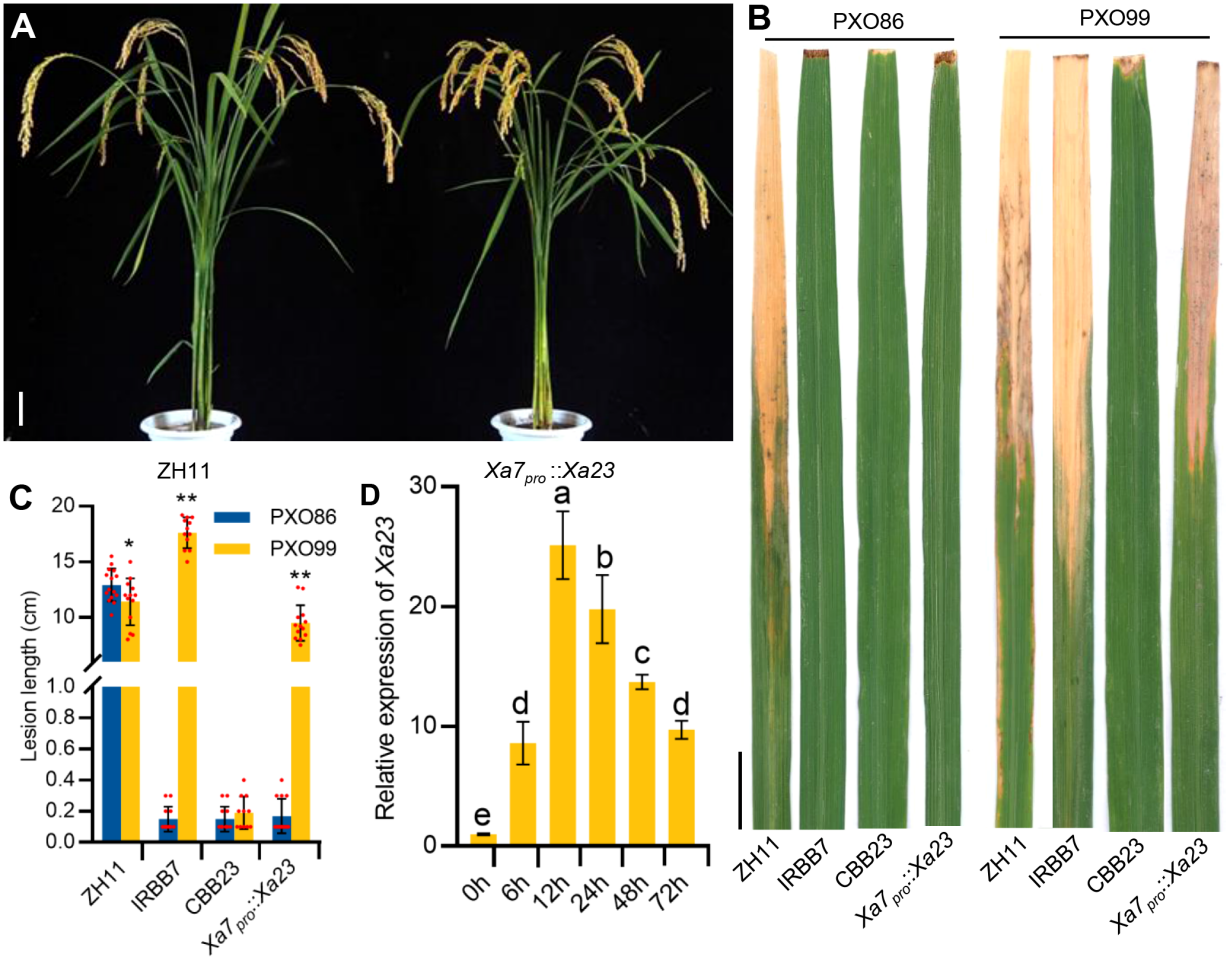
The resistance and expression characteristics of *Xa7_pro_::Xa23* transgenic plants. **(A)** Phenotypes of ZH11 and *Xa7_pro_::Xa23* transgenic plants at mature stage. Bar = 10 cm. **(B)** Lesions of ZH11, IRBB7, CBB23, and the *Xa7_pro_::Xa23* transgenic plants inoculated with *Xoo* strains PXO86 and PXO99. Photographs were taken 2 weeks after inoculation with *Xoo* strains. Bar = 2 cm. **(C)** Lesion lengths (cm) of ZH11, IRBB7, CBB23, and the *Xa7_pro_::Xa23* transgenic plants. Data are shown as means ± SD; significance analysis was conducted using a two-tailed Student’s *t*-test. **(D)** The induced expression characteristics of *Xa23* gene in the *Xa7_pro_::Xa23* transgenic plants. Leaves were sampled at 0 hours, 6 hours, 12 hours, 24 hours, 48 hours, and 72 hours after inoculated with PXO86. Data are shown as means ± SD; significance analysis was conducted using ordinary one-way ANOVA with least significant difference (LSD) multiple comparisons test. * represents significance differences exist at p < 0.05; ** represents significance differences exist at p < 0.01.

To further verify whether the executor *R* gene driven by the *Xa7* promoter can enhance the disease resistance of rice plants, the mentioned construct *Xa7_pro_::Xa23* was transformed into a BB-susceptible rice cultivar Zhonghua11 (ZH11). Twenty-five stable transgenic T_2_ lines were obtained and used for the analysis of the BB resistance (**Figure 3A**). The transgenic recipient ZH11 was highly susceptible to BB, while the transgenic plants with *Xa7_pro_::Xa23* exhibited a similar disease resistance spectrum of *Xa7*, instead of *Xa23*. The *Xa7_pro_::Xa23* transgenic plants were resistant to the *Xa7*-incompatible strain PXO86 and susceptible to the *Xa7*-compatible strain PXO99, while rice cultivar CBB23 containing the native *Xa23* gene was resistant to both stains (**Figures 3B, C**). Additionally, the expression of *Xa23* gene in those transgenic lines was significantly induced at different time points after PXO86 inoculation (**Figure 3D**). The above results showed that the executor *R* gene driven by the promoter of *Xa7* gene enhanced host resistance to BB and indicated that the disease resistance characteristics of the executor *R* genes mainly depend on their promoters.

### Expression of the *Xa7* gene is induced locally by its incompatible *Xoo* strain

**Figure 4:**
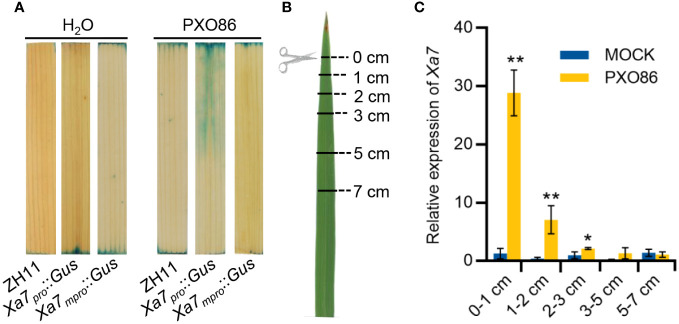
Induction expression characteristics of the *Xa7* gene. **(A)** The GUS staining results of leaves from transgenic plants *Xa7_pro_::Gus* and *Xa7_mpro_::Gus*. Leaves were stained after inoculated with H_2_O and *Xoo* strain PXO86 for 24 hours. **(B)** Leaves of IRBB7 were inoculated with PXO86 by clipping the leaf tips, and the infected leaves were divided into five different regions away from the clipping site (0–1 cm, 1–2 cm, 2–3 cm, 3–5 cm, and 5–7 cm). **(C)** Expression of the *Xa7* gene in different leaf regions after inoculation with PXO86 and H_2_O for 24 hours. Gene expression was measured by qRT-PCR, and data are presented as the means of three replicates ± SD; significance analysis was conducted using a two-tailed Student’s *t*-test; * represents significance differences exist at *p* < 0.05; ** represents significance differences exist at *p* < 0.01.

To explore the mechanism of *Xa7* gene-mediated disease resistance, the expression pattern of the *Xa7* gene after inoculation with *Xoo* strains was analyzed. A native promoter of the *Xa7* gene (abbreviated as *Xa7_pro_*) and a mutated promoter of the *Xa7* gene (abbreviated as *Xa7_mpro_*) with two base deletions in the EBE_AvrXa7_ sequence were constructed upstream of the *Gus* gene and obtained corresponding transgenic rice plants designated as *Xa7_pro_::Gus* and *Xa7_mpro_::Gus*. After inoculation with the *Xa7* incompatible *Xoo* strain PXO86, which contains an AvrXa7, GUS activity was significantly detected in leaves of the *Xa7_pro_::Gus* transgenic plants, and it was localized around the infection site, while no induced GUS activity was detected in the leaves of the *Xa7_mpro_::Gus* transgenic plants (**Figure 4A**).

Additionally, leaves from rice cultivar IRBB7 that contain the *Xa7* gene were inoculated with PXO86 by clipping the leaf tips. After 24 hours, the infected leaves were collected and divided into five different regions based on distance from the clipping site (0–1 cm, 1–2 cm, 2–3 cm, 3–5 cm, and 5–7 cm) (**Figure 4B**). The expression level of the *Xa7* gene was then analyzed *via* RT-qPCR. The results showed that expression of the *Xa7* gene was strongly induced near the infection site of the leaves (<1 cm) and gradually decreased as the distance increased (**Figure 4C**). These results suggested that the *Xa7* gene was locally induced in the host cells by *Xoo* infection.

### Activation of the *Xa7* gene inhibits the growth of incompatible *Xoo* strain

**Figure 5:**
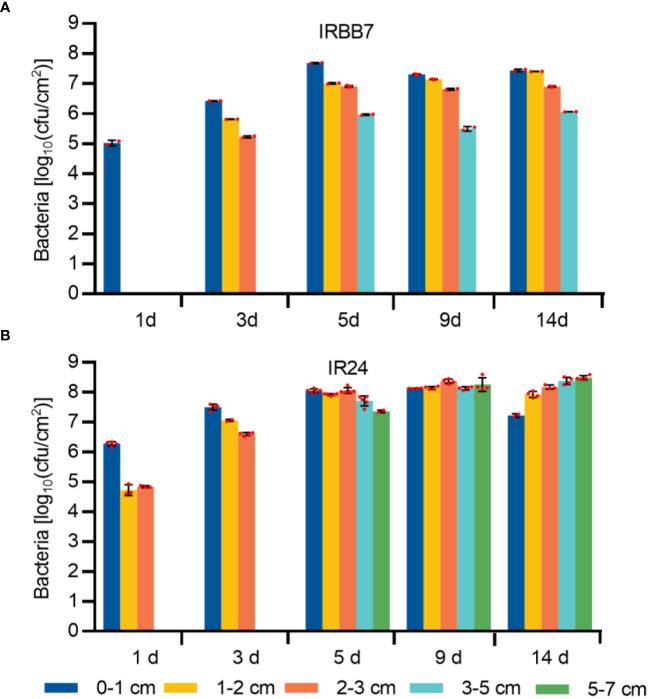
Growth quantity of *Xoo* strains in the leaves of IRBB7 and IR24 after inoculation with PXO86. **(A, B)** Population number of *Xoo* strains after inoculation with PXO86 for 1 day, 3 days, 5 days, 9 days, and 14 days in IRBB7 and IR24, respectively. Leaves were sampled from the regions 0–1 cm, 1–2 cm, 2–3 cm, 3–5 cm, and 5–7 cm after inoculated with PXO86.

As the induced expression of the *Xa7* gene is localized, it is likely that the pathogen would be controlled locally in the host. To investigate this, the leaves of IR24 and its near-isogenic line IRBB7 were inoculated with PXO86 *via* leaf-tip clipping, and the growth of *Xoo* strains was analyzed in the five different regions of the inoculated leaves (**Figure 4B**) at 1 day, 3 days, 5 days, 9 days, and 14 days after inoculation (DAI) (**Figures 5A, B**). At 1 DAI, the growth of *Xoo* was only detected in the 0–1-cm region of the IRBB7 leaves, whereas it was detected in the 0–3-cm region of the IR24 leaves. Subsequently, the growth of *Xoo* spread on the leaves from the tips of both IRBB7 and IR24. However, the pathogen was limited to the 0–5-cm region of IRBB7 from 5 DAI, while it had spread to areas beyond 7 cm in IR24. Moreover, the amount of *Xoo* strains in IRBB7 was at least 10-fold lower than that in IR24 over 0–1-cm regions. These results indicated that the induced expression of the *Xa7* gene could retard and restrict the growth of *Xoo* strains.

### The induced expression of the *Xa7* gene enhances resistance of rice plants to compatible strains

**Figure 6:**
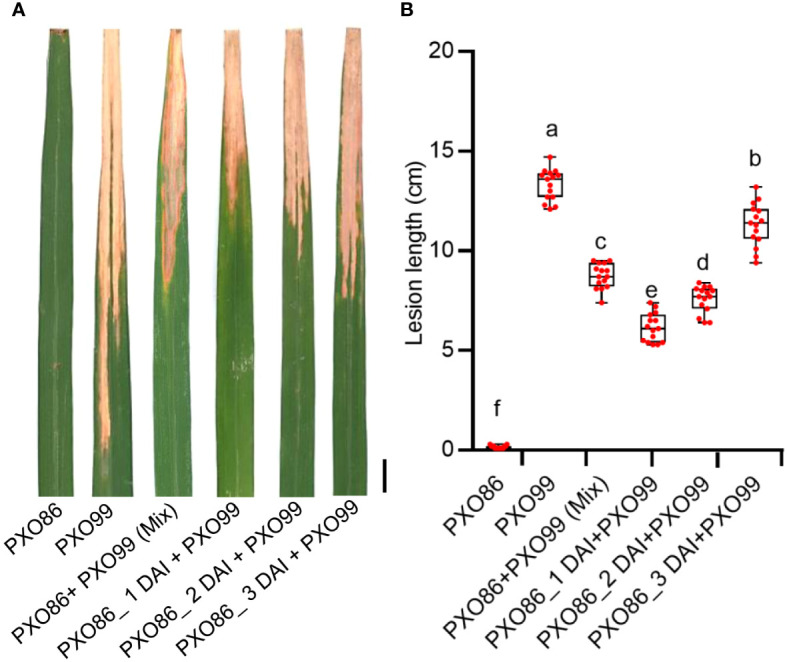
Evaluation of disease resistance to compatible strain PXO99 after induced expression of the *Xa7* gene. **(A)** The lesion phenotype of IRBB7 after inoculation with different races of *Xoo* strains for 2 weeks. Bar = 1 cm. **(B)** Statistics of lesion length in panel **(A)** From left to right, they represent leaves of IRBB7 infected with PXO86, PXO99, PXO86+PXO99 (Mix), and combined inoculation of PXO99 after inoculated with PXO86 for 1 day, 2 days, and 3 days. Data are shown as means ± SD; significance analysis was conducted using ordinary one-way ANOVA with least significant difference (LSD) multiple comparisons test.

As the induced expression of *Xa7* gene inhibited the growth of incompatible strain PXO86, we hypothesized that the activated expression of the *Xa7* gene may also enhance the host’s defense against compatible *Xoo* strains. To test this hypothesis, a frequently used *Xoo* strain PXO99, which does not contain an AvrXa7 and cannot activate the expression of the *Xa7* gene, was selected as a compatible strain of the *Xa7* gene (Chen et al., 2021). First, the leaves of IRBB7 were infected with the incompatible strain PXO86 to activate the expression of the *Xa7* gene and then further inoculated with the compatible strain PXO99 by clipping closely to the PXO86-infected site at 1 day, 2 days, and 3 days after PXO86 inoculation (<0.5 cm). The lesion lengths on leaves were measured 2 weeks later after *Xoo* inoculation. The results showed that the lesions of leaves inoculated with combined PXO86 and PXO99 strains were significantly shorter than those only inoculated with PXO99 (**Figures 6A, B**). To further verify that the observed effect was solely due to AvrXa7-mediated induction of *Xa7*, the leaves of IRBB7 were infected with PXO99*^avrXa7^*, PXO99, and combined strains (PXO99*^avrXa7^* and PXO99 mixed). The lesion lengths were measured 2 weeks later after *Xoo* inoculation (**Supplementary Figure S2**), and the results were consistent with those shown in **Figure 6**. Thus, the induced expression of the *Xa7* gene could significantly increase the resistance of host plants to the compatible strains.

### *Xa7* gene mediates transcriptional reprogramming during disease resistance

**Figure 7:**
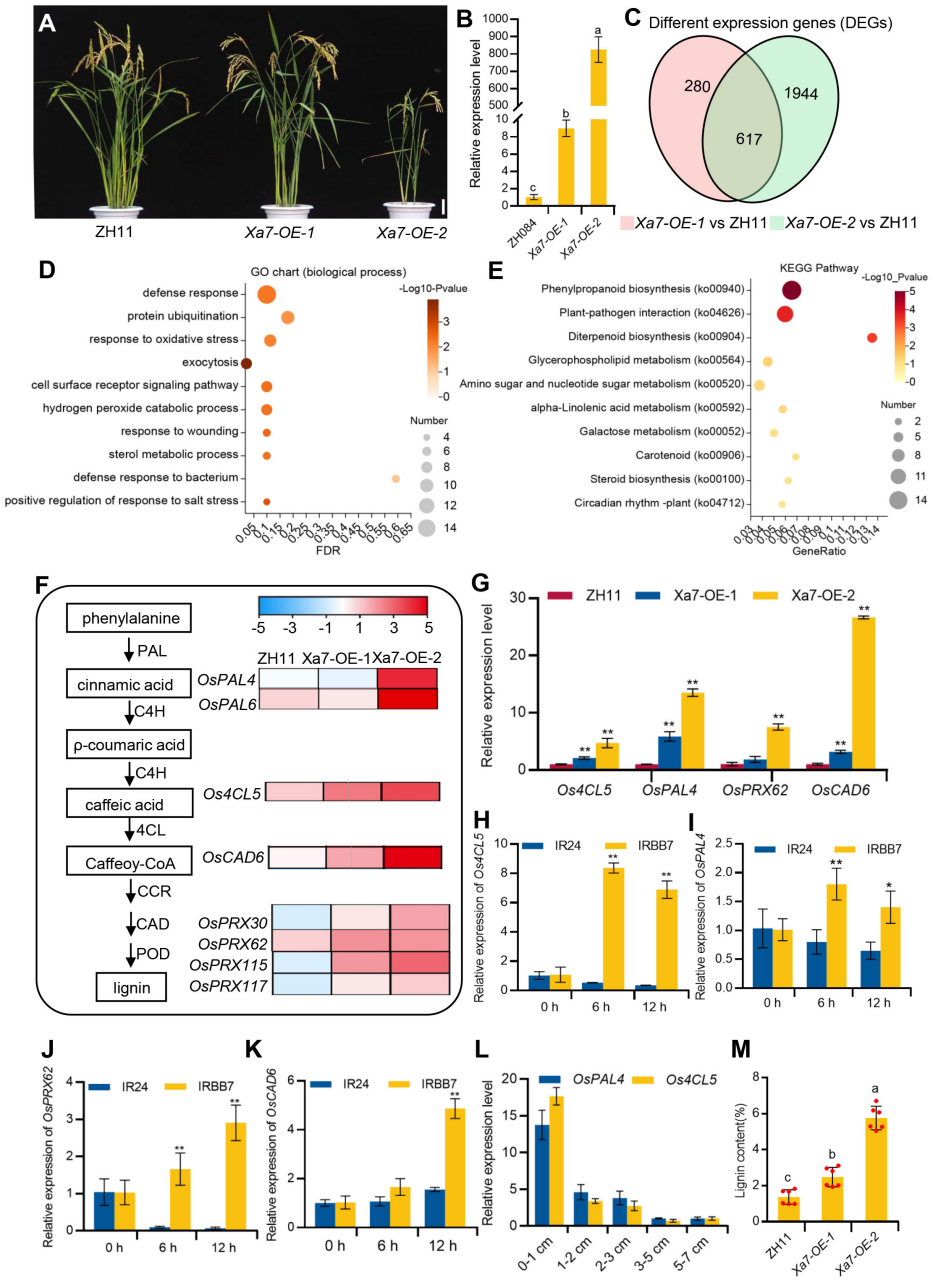
Transcriptome analysis and verification of the *Xa7-*constitutive expression lines. **(A)** Phenotypic characterization of ZH11, *Xa7-OE-1*, and *Xa7-OE-2*. Bar = 10 cm. **(B)** qRT-PCR analysis of the relative transcript levels of the *Xa7* gene in ZH084, *Xa7-OE-1*, and *Xa7-OE-2* with the absence of *Xoo*. **(C)** Venn diagram illustrating the number of differentially expressed genes (DEGs) identified in *Xa7-OE-1* and *Xa7-OE-2* compared to ZH11. **(D)** Gene Ontology (GO) enrichment analysis of the DEGs in *Xa7-OE-1* and *Xa7-OE-2* when compared to ZH11. **(E)** The Kyoto Encyclopedia of Genes and Genomes (KEGG) pathways enrichment of the upregulated DEGs between *Xa7-OE-1* and *Xa7-OE-2* when compared to ZH11. The value on the right side of the column indicates the reliability of the enrichment degree of the pathways (*p*-value). **(F)** The heat map of DEGs associated with the phenylpropanoid pathway. Log values of the FPKM of DEGs were used for the display of the heat map. **(G)** Verification of the relative expression levels of DEGs in rice plants ZH11, *Xa7-OE-1*, and *Xa7-OE-2*. Data are shown as means (three independent replicates) ± SD. (**H**–**K**) The expression levels of *Os4CL5*, *OsPAL4*, *OsPRX62*, and *OsCAD6* in IRBB7 and IR24 at different time points after inoculation with PXO86, respectively. Data are shown with means ± SD. **(L)** The expression of *OsPAL4* and Os4CL5 in different leaf regions 24 hours after IRBB7 inoculated with PXO86. **(M)** The quantitative determination of lignin content in rice plants ZH11, *Xa7-OE-1*, and *Xa7-OE-2*. In panels **(B, G–K, M**), statistical significance was determined by ordinary one-way ANOVA with least significant difference (LSD) multiple comparisons test. * represents significance differences exist at p < 0.05; ** represents significance differences exist at p < 0.01.

To further explore the molecular basis of cell death and defense response mediated by the *Xa7* gene, transgenic rice plants constitutively expressing the *Xa7* gene that was constructed in our previous study were applied (Chen et al., 2021). Two different lines, named *Xa7-OE-1* and *Xa7-OE-2*, were selected. Compared to the wild-type control ZH11, *Xa7-OE-1* plants display normal phenotypes and have low expression levels of the *Xa7* gene, while the growth and development of the *Xa7-OE-2* plants are obviously inhibited and have significantly higher expression of the *Xa7* gene (**Figures 7A, B**). RNA-seq analysis was performed on both transgenic lines and the wild-type ZH11. Compared to ZH11, 897 and 2,561 DEGs were detected in *Xa7-OE-1* and *Xa7-OE-2*, respectively, and among them, 617 DEGs were shared by the two transgenic lines (**Figure 7C**), in which 491 genes were collectively upregulated and 87 genes were downregulated (**Supplementary Table S1**, **S2**).

The Gene Ontology (GO) enrichment of the upregulated DEGs showed that genes involved in biological processes and defense response were prevalent (**Figure 7D**), such as the transcription factor OsWRKY19, which can activate the transcription of *PR* gene and play an important role in the defense response against pathogens (Du et al., 2021). To further explore *Xa7*-mediated pathways, KEGG enrichment analysis of the DEGs was performed, and the most enriched pathway was phenylpropanoid biosynthesis (ko00940) (**Figure 7E**). It has been reported that this pathway is involved in lignin biosynthesis and plays a key role in plant protection under biotic stress (Hamberger et al., 2007; Vogt, 2010). Enzymes such as phenylalanine ammonia-lyase (PAL), 4-coumarate–CoA ligase (4CL), cinnamoyl-CoA reductase (CCR), and cinnamyl alcohol dehydrogenase (CAD) are involved in the biosynthesis of monolignol from phenylalanine (Vanholme et al., 2010; Tianpei et al., 2015), and then laccases (Lac) or peroxidases (POD) oxidize monolignols to monolignol radicals in the cell wall, which subsequently combine in a combinatorial fashion to form lignin (Miedes et al., 2014). In the *Xa7-OE* lines, genes encoding the enzyme involved in lignin biosynthesis, such as PAL (*OsPAL4* and *OsPAL6*), 4CL (*Os4CL5*), CAD (*OsCAD6*), and POD (*OsPRX30*, *OsPRX62*, *OsPRX115*, and *OsPRX117*), were found to be significantly upregulated (**Figure 7F**).

The transcription levels of selected genes, including *OsPAL4*, *Os4CL5*, *OsPRX62*, and *OsCAD6*, were verified *via* qRT-PCR, and the results were consistent with the RNA-seq data (**Figure 7G**). Furthermore, the expression of these genes was significantly induced in the leaves of IRBB7 but not in IR24 after inoculation with the PXO86 strain (**Figures 7H–K**). Moreover, qRT-PCR was selected to detect the expression level of two lignin-related genes in the different regions of IRBB7 after inoculation with PXO86 24 hours, and the results showed that the expression pattern was similar to that of the *Xa7* gene. Subsequently, quantitative determination of lignin content in the leaves from ZH11, *Xa7-OE-1*, and *Xa7-OE-2* proved that with the increased expression of *Xa7*, the lignin content was significantly enriched (**Figure 7M**). The above results suggested that the executor *R* gene *Xa7* may enhance the resistance of rice plants to *Xoo* pathogens by increasing lignin synthesis.

## Discussion

The identification and cloning of executor *R* genes from rice and pepper that can specifically trap TALEs of pathogens and trigger defense reactions in host plants have revolutionized our understanding of plant–pathogen interactions (Ji et al., 2022b; Nowack et al., 2022). Among the six executor *R* genes cloned to date, *Xa7* is a newly identified executor *R* gene of rice that contains a putative AvrXa7-target sequence EBE_AvrXa7_ in its promoter (Chen et al., 2021; Luo et al., 2021; Wang et al., 2021). Here, we experimentally confirmed that AvrXa7 can directly bind to the EBE_AvrXa7_, suggesting that *Xa7* can be activated by the binding of AvrXa7 to its promoter *via* EBE_AvrXa7_ (**Figure 1**).

In fact, the specific recognition between the EBEs of target genes in the host plant and the RVDs of TALEs plays a critical role in determining disease susceptibility or resistance in plants. Variations in the EBE sequences of *S* genes in plants decreased their susceptibility to pathogen attack (Antony et al., 2010). Conversely, the TALE-dependent *R* genes of plants have evolved to specifically interact with TALEs to activate immunity. To date, four executor genes, *Xa7*, *Xa10*, *Xa23*, and *Xa27*, have been cloned from rice, which confer race-specific disease resistance against *Xoo* strains carrying TALEs AvrXa7, AvrXa10, AvrXa23, and AvrXa27, respectively (Ji et al., 2022b). In this study, we proved that the disease resistance spectrum of *Xa7* is largely determined by its promoter, as the executor *R* gene *Xa23* driven by the *Xa7* promoter was found to confer similar disease resistance characteristics to *Xa7* rather than *Xa23* (**Figure 3**). Promoter modification of *R* genes has been proposed as an effective strategy for engineering novel recognition specificity between TALEs and EBEs to gain broad-spectrum resistance to pathogens (Li et al., 2016). For instance, in the transgenic rice plants, six EBE sequences specifically recognized by cognate TALEs of PthXo1, PthXo6, Tal9a, Tal4c, Tal2g, and Tal4a were introduced into the promoter of *Xa27* gene to achieve broad-spectrum resistance to both *Xoo* and *Xoc* (*X. oryzae* pv. *oryzicola*) strains (Hummel et al., 2012). Similarly, an additional 17-bp EBE inserted into the promoter of *Xa23* in rice plants also specifically trapped conserved TALEs from multiple *Xoc* strains, providing an alternative strategy to effectively utilize the executor *R* genes in disease resistance (Ji et al., 2022a). In the future, as more *R* genes and TALEs are identified, pyramiding the EBEs of major TALEs from pathogens, such as AvrXa7, AvrXa23, PthXo3, and Tal2g, will be a promising strategy for breeding crops with broad-spectrum disease resistance.

In this research, we were surprised to find that *Xa7* was locally activated in infected leaves by the incompatible strain PXO86 (**Figure 4**), and compared to the rice variety IR24, the bacteria grew to similar levels in the infiltration area and significantly deceased outside of the infection site, finally limited inside of the 5-cm region in IRBB7 (**Figure 5**). This response was similar to the localized acquired resistance (LAR), which did not suppress pathogen growth at the local site but rather halted pathogen propagation into adjacent regions (Jacob et al., 2023). The mechanism of LAR is distinct from that of systemic acquired resistance (SAR), which primes defense in distal tissues to prevent secondary infections (Ross, 1961). Contrary to SAR, LAR is acute and short-lasting, and the HR triggered by an elicitor was found to activate defense in the surrounding cells without direct contact with the elicitor (Dorey et al., 1997). Moreover, *Xa7* expression appeared to occur earlier than the onset of cell death (**Figure 4**). This raises a possibility that complete cell death may not be necessary to generate cell non-autonomous danger signals that activate LAR. Additionally, both the induced and constitutive expression of *Xa7* gene enhanced the resistance of rice plants to *Xa7*-compatible strain PXO99 (**Figure 6**; Chen et al., 2021), indicating that the LAR activated by the *executor* genes has no race specificity. An open question is to identify the immunogenic molecules triggering LAR. Candidates like ROS or calcium signaling released from infection cells are worth investigating.

Groundbreaking research on the *Xa10* provided exciting insights into how *Xa7* initiated immune signaling. Similar to XA7, XA10 was a compact protein featuring four transmembrane α-helices and localized to the endoplasmic reticulum (ER) membrane of plants, which was coincidental with the ER Ca^2+^ depletion and the onset of XA10-induced cell death (Tian et al., 2014). The ER is one of the important intracellular Ca^2+^ stores in eukaryotic cells, and the reduction of ER Ca^2+^ levels can induce the unfolded protein response, an evolutionarily conserved stress response that can trigger apoptosis (Shore et al., 2011). Additionally, the formation of hexamers of XA10 on the ER membrane was reinforced by the WTS protein, which formed pentameric architecture with a central pore and functioned as a new type of Ca^2+^ permeable cation-selective channel on the ER membrane (Wang et al., 2023). Based on the results obtained in the study of *Xa10* and *WTS*, we propose that *Xa7* may form a calcium channel on the ER membrane and transport Ca^2+^ from the ER to the cytosolic when the pathogens are present; however, this hypothesis remains to be validated further.

In order to investigate the disease resistance mechanism conferred by the *Xa7* gene, RNA-seq was performed. In the present study, several genes encoding key enzymes of lignin synthesis were induced in both the *Xa7*-overexpression lines and the *Xa7*-containing rice variety IRBB7 after *Xoo* inoculation, resulting in the accumulation of lignin (**Figure 7**). Lignin is the second most abundant component in the cell walls of vascular tissues (Neutelings, 2011) and is often rapidly deposited through the induction of lignin biosynthesis genes in responses to pathogen infection in plants (Malinovsky et al., 2014; Yoon et al., 2015). The lignin-mediated vascular defense was also used by other race-specific *R* genes against vascular pathogens in the rice–*Xoo* pathosystem. For example, the Xa*21*-mediated *Xoo* resistance was related to lignin accumulation (Shamsunnaher et al., 2020). Similarly, *Xa10* stimulated the accumulation of lignin-like phenolic compounds (Reimers and Leach, 1991). Based on these findings, it is possible to speculate that *Xa7* may enhance the resistance to *Xoo* by increasing the biosynthesis of lignin, which could change the mechanical strength of the cell wall and enhance resistance against *Xoo*. This raises the following question: how does *Xa7* gene activate lignin-based vascular-specific immune responses upon perceiving *Xoo* in rice? This will be worthy of further investigation. All in all, the discovery of the disease resistance mechanism of *Xa7* opens the door to investigating how the other *executor* genes confer resistance to *Xoo*.

## Data availability statement

The datasets presented in this study can be found in online repositories. The names of the repository/repositories and accession number(s) can be found below: BioProject, PRJNA954312.

## Author contributions

LH: Writing – original draft, Writing – review & editing. PL: Funding acquisition, Writing – original draft, Writing – review & editing. LM: Writing – review & editing. HL: Writing – review & editing. TB: Writing – review & editing. XC: Writing – review & editing, Funding acquisition. BM: Writing – review & editing, Funding acquisition.
