# Supplementary material for: Disease resistance features of the executor R gene Xa7 reveal novel insights into the interaction between rice and Xanthomonas oryzae pv. oryzae
Source: Front Plant Sci. 2024 Apr 3;15:1365989. doi: 10.3389/fpls.2024.1365989 (PMC11021754; doi:10.3389/fpls.2024.1365989)
Supplement: Supplementary file 1 [file DataSheet_1.pdf]

Supplement Figure 1

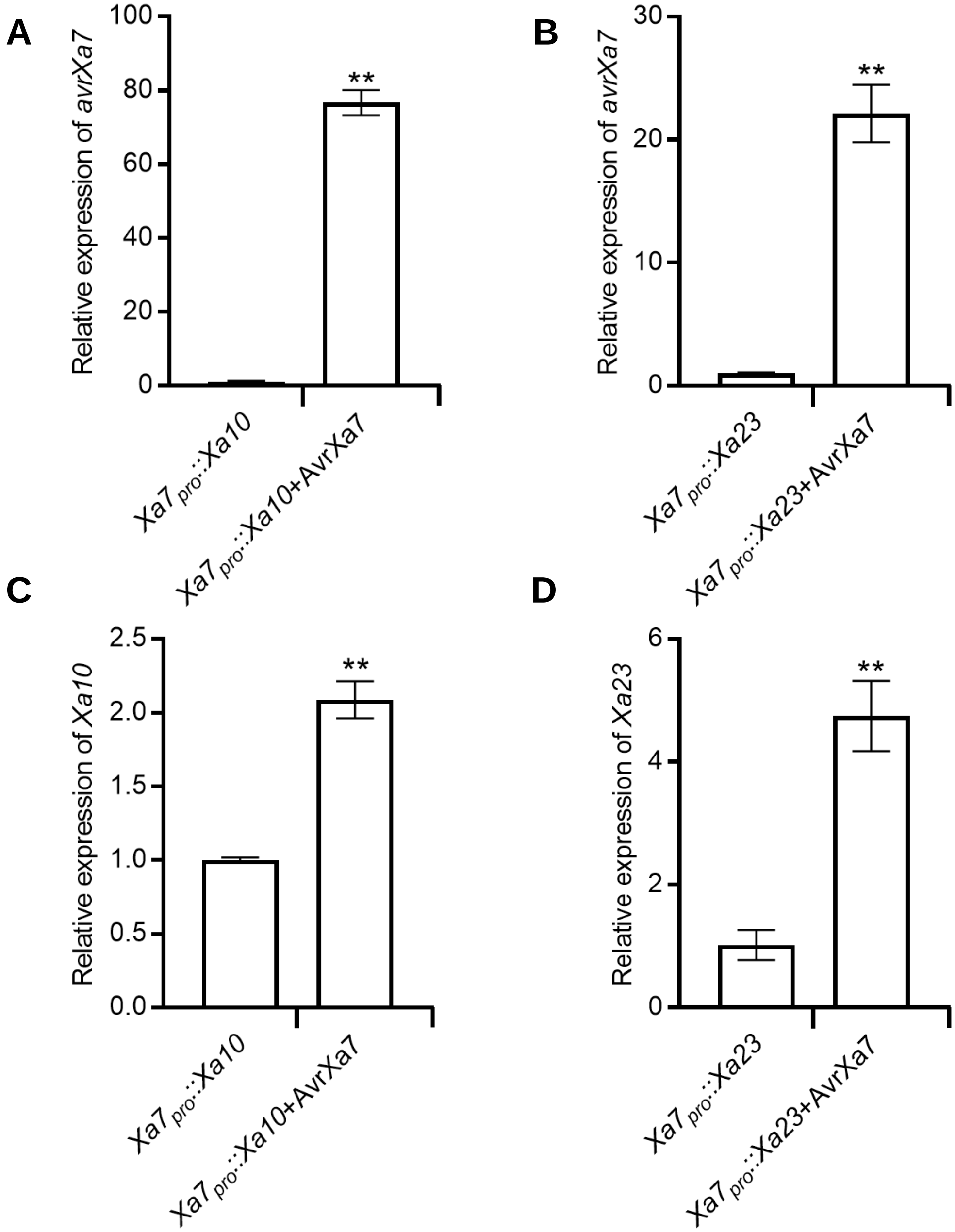

Supplement Fig.1 Relative expression of *avrXa7* or *executor* genes in leaf tissues of *N.benthamiana* at 48 HAI. **A-B,** qRT-PCR analysis of the relative transcript levels of the *avrXa7* gene in tobacco. Transforming $Xa7_{pro}$*::executor* (*Xa10* or *Xa23*) without *35S::avrXa7* as the negative control, respectively. **C-D,** The expression levels of *executor* genes co-injected with the *35S::AvrXa7* in tobacco. The average expression of negative control was set as “1” and the data were presented as means ± SD. Asterisks represent statistical significance as *P < 0.05, **P < 0.01, respectively.

Supplement Figure 2

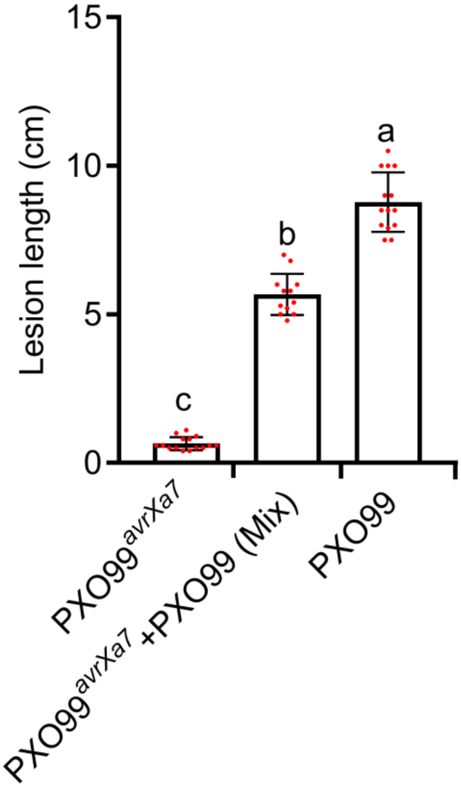

Supplement Fig.2 Evaluation of disease resistance to compatible strain PXO99 with *Xa7* induction. Statistics of lesion length of IRBB7 infected with PXO99^{avrXa7}, PXO99^{avrXa7} + PXO99 (Mix), and PXO99, respectively. Data were shown as means ± SD, significance analysis was conducted by ordinary one-way ANOVA with LSD's multiple comparisons test.
