# Supplementary material for: Disease resistance features of the executor R gene Xa7 reveal novel insights into the interaction between rice and Xanthomonas oryzae pv. oryzae
Source: Front Plant Sci. 2024 Apr 3;15:1365989. doi: 10.3389/fpls.2024.1365989 (PMC11021754; doi:10.3389/fpls.2024.1365989)
Supplement: Supplementary file 2 [file Table_1.docx]

**Sequences of fragments in constructs used for transformation**

> fragments of the RVDs of AvrXa7 used in vector construction

TGAAGCAACTTAAAGTTATCAGGCATGCATGGCGCAATGCACTGACGGGTGCCCCCCTGAACCTGACCCCGGACCAAGTGGTGGCCATCGCCAGCAATATTGGCGGCAAGCAGGCGCTGGAGACGGTACAGCGGCTGTTGCCGGTGCTGTGCCAGGACCATGGCCTGACCCCGGACCAGGTCGTGGCCATCGCCAGCCATGGCGGCGGCAAGCAGGCGCTGGAGACGGTGCAGCGGCTGTTGCCGGTGCTGTGCCAGGACCATGGCCTGACCCCGGACCAGGTGGTGGCCATCGCCAGCAATATTGGCGGCAAGCAGGCGCTAGAGACGGTGCAGCGGCTGTTGCCGGTGCTGTGCCAGGCCCATGGCCTGACCCCGGACCAGGTCGTGGCCATCGCCAGCAATATTGGCGGCAAGCAGGCGCTGGAGACGGTGCAGCGGCTGTTGCCGGTGCTGTGCCAGGACCATGGCCTGACCCCGGCCCAGGTGGTGGCCATCGCCAGCAATAGTGGCGGCAAGCAGGCGCTGGAGACGGTGCAGCGGCTGTTGCCGGTGCTGTGCCAGGACCATGGCCTGACCCCGGACCAAGTCGTGGCCATCGCCAGCCACGATGGCGGCAAGCAGGCGCTGGAGACGGTGCAGCGGCTGTTGCCGGTGCTGTGCCAGGACCATGGCCTGACCCCGGACCAGGTCGTGGCCATCGCCAACAATAACGGCGGCAAGCAGGCGCTGGAGACGCTGCAGCGGCTGTTGCCGGTGCTGTGCCAGGACCATGGCCTGACCCCGGACCAAGTGGTGGCCATCGCCAGCCACGATGGCGGCAAGCAGGCGCTGGAGACGGTGCAGCGGCTGTTGCCGGTGCTGTGCCAGGACCATGGCCTGACCCCGGACCAGGTGGTGGCCATCGCCAGCCACGATGGCGGCAAGCAGGCGCTGGAGACGGTGCAGCGGCTGTTGCCGGTGCTGTGCCAGGACCATGGCCTGACCCCGGCCCAAGTGGTGGCCATCGCCAGCCACGATGGCGGCAAGCAGGCGCTGGAGACGGTGCAGCGGCTGTTGCCGGTGCTGTGCCAGGACCATGGCCTGACCCCGGACCAGGTGGTGGCCATCGCCAGCAATAGCGGCGGCAAGCAGGCGCTGGAGACGGTACAGCGGCTGTTGCCGGTGCTGTGCCAGGACCATGGACTGACCCCGGACCAGGTCGTGGCCATCGCCAGCAATGGCGGCAAGCAGGCGCTGGAGACGGTACAGCGGCTGTTGCCGGTGCTGTGCCAGGACCATGGCCTGACCCCGGACCAGGTCGTGGCCATCGCCAGCAATGGCGGCAAGCAGGCGCTGGAGACGGTGCAGCGGCTGTTGCCGGTACAGCGGCTGTTGCCGGTGCTGTGCCAGGACCATGGCCTGACCCAGGACCAGGTGGTGGCCATCGCCAGCCACGATGGCGGCAAGCAGGCGCTGGAGACGGTGCAGCGGCTGTTGCCGGTGCTGTGCCAGGACCATGGCCTGACCCCGGACCAAGTGGTGGCCATCGCCAGCCACGATGGCGGCAAACAGGCGCTGGAGACGGTGCAGCGGCTGTTGCCGGTGCTGTGCCAGGACCATGGCCTGACCCCGGACCAGGTGGTGGCCATCGCCAGCAATAGTGGCGGCAAGCAGGCGCTGGAGACGGTGCAGCGGCTGTTGCCGGTGCTGTGCCAGGACCATGGCCTGACCCCGGACCAAGTGGTGGCCATCGCCAGCAATAGTGGCGGCAAGCAGGCGCTGGAGACGGTGCAGCGGCTGTTGCCGGTGCTGTGCCAGGACCATGGCCTGACCCCGGACCAGGTGGTGGCCATCGCCAGCAATAACGGCGGCAAGCAGGCGCTGGAGACGGTGCAGCGGCTGTTGCCGGTGCTGTGCCAGGACCATGGCCTGACCCCGGACCAGGTCGTGGCCATCGCCAACAATAACGGCGGCAAGCAGGCGCTGGAGACGGTGCAGCGGCTGTTGCCGGTGCTGTGCCAGGACCATGGCCTGACCCCGGCGCAGGTGGTGGCCATCGCCAGCAATATTGGCGGCAAGCAGGCGCTGGAGACGGTGCAGCGGCTGTTGCCGGTGCTGTGCCAGGACCATGGCCTGACCCTGGACCAGGTGGTGGCCATTGCCAGCAATGGCGGCAGCAAACAGGCGCTAGAGACGGTGCAGCGGCTGTTGCCGGTGCTGTGCCAGGACCATGGCCTGACCCCGGACCAAGTGGTGGCCATCGCCAACAATAACGGCGGCAAGCAGGCGCTGGAGACGGTGCAGCGGCTGTTGCCGGTGCTGTGCCAGGACCATGGCCTGACCCCGGACCAGGTCGTGGCCATCGCCAGCAATATTGGCGGCAAGCAGGCGCTGGAGACGGTGCAGCGGCTGTTGCCGGTGCTGTGCCAGGACCATGGCCTGACCCTGGACCAGGTGGTGGCCATCGCCAGCAATGGCGGCAAGCAGGCGCTGGAGACGGTGCAGCGGCTGTTGCCGGTGCTGTGCCAGGACCATGGCCTGACCCCGAACCAGGTGGTGGCCATCGCCAGCAATAGTGGCGGCAAGCAGGCGCTGGAGACGGTGCAGCGGCTGTTGCCGGTGCTGTGCCAGGACCATGGCCTGACCCCGAACCAGGTGGTGGCCATCGCCAGCAATGGCGGCAAGCAGGCGCTGGAGAGCATTGTTGCCCAGTTATCTCGCCCTGATCCGGCGTTGGCCGCGTTGACCAACGACCACCTCGTCGCCTTGGCCTGCCTCGGCGGACGTCCTGCCCTGGATGCAGTGAAAAAGGGATTGCCGCACGCGCCGGAATTGATCAGAAGAATCAATCGCCGCATTCCCGAACGCACGTCCCATCGCGTTCCCGACCTCGCGCACGTGGTTCGCGTGCTTGGTTTTTTCCAGAGCCACTCCCACCCAGCGCAAGCATTCGATGACGCCATGACGCAGTTCGAGATGAGCAGGCACGGCTTGGTACAGCTCTTTCGCAGAGTGGGCGTCACCGAATTCGAAGCCCGCTACGGAACGCTCCCCCCAGCCTCGCAGCGTTGGGACCGTATCCTCCAGGCATCAGGGATGAAAAGGGCCAAACCGTCCCCTACTTCAGCTCAAACACCGGATCAGGCGTCTTTGCATGCGACGTCGATCTAACTGACTAGC

> fragments of the *Xa7*-EBE_AvrXa7_ and *SWEET14*-EBE_AvrXa7_ used in vector construction

*Xa7*-EBE_AvrXa7_:

TATAACCCCCCCCCCCCCAGATAACCATATAACCCCCCCCCCCCCAGATAACCATATAACCCCCCCCCCCCCAGATAACCA

*SWEET14*-EBE_AvrXa7_:

TATAAACCCCCTCCAACCAGGTGCTAATATAAACCCCCTCCAACCAGGTGCTAATATAAACCCCCTCCAACCAGGTGCTAA

> fragments of *35S::Xa7* used in vector construction

GTCCCCAGATTAGCCTTTTCAATTTCAGAAAGAATGCTAACCCACAGATGGTTAGAGAGGCTTACGCAGCAGGTCTCATCAAGACGATCTACCCGAGCAATAATCTCCAGGAAATCAAATACCTTCCCAAGAAGGTTAAAGATGCAGTCAAAAGATTCAGGACTAACTGCATCAAGAACACAGAGAAAGATATATTTCTCAAGATCAGAAGTACTATTCCAGTATGGACGATTCAAGGCTTGCTTCACAAACCAAGGCAAGTAATAGAGATTGGAGTCTCTAAAAAGGTAGTTCCCACTGAATCAAAGGCCATGGAGTCAAAGATTCAAATAGAGGACCTAACAGAACTCGCCGTAAAGACTGGCGAACAGTTCATACAGAGTCTCTTACGACTCAATGACAAGAAGAAAATCTTCGTCAACATGGTGGAGCACGACACACTTGTCTACTCCAAAAATATCAAAGATACAGTCTCAGAAGACCAAAGGGCAATTGAGACTTTTCAACAAAGGGTAATATCCGGAAACCTCCTCGGATTCCATTGCCCAGCTATCTGTCACTTTATTGTGAAGATAGTGGAAAAGGAAGGTGGCTCCTACAAATGCCATCATTGCGATAAAGGAAAGGCCATCGTTGAAGATGCCTCTGCCGACAGTGGTCCCAAAGATGGACCCCCACCCACGAGGAGCATCGTGGAAAAAGAAGACGTTCCAACCACGTCTTCAAAGCAAGTGGATTGATGTGATATCTCCACTGACGTAAGGGATGACGCACAATCCCACTATCCTTCGCAAGACCCTTCCTCTATATAAGGAAGTTCATGGCGGCCGCTGATCATCCTGATCGTATGCCCGTTGCAGTTGCAGGCTTGCGCCACCATTACGCCTTCCCTGCAAACCTTCGCCCCGCCGCTCGACTGCTGACCGTCAACTCCGGCGTCTTCCTCATCTCCACCGCCGGGGCCATCGTCCTCGTCCACACCGCCGGTAACCCACCCGCCATCGACAACGATCCAGCCTACGCCTTGGTCGCATTCGTGCTCTTCCTCCTCGGAATCTGGCTCATGTCTATTGCCCTCGTCGCCGACCAGTTCCCGCGCGCCGCTGGGGTCGCCGTGGCCATTGCCAGGGCGCTGCAGGATTACCTCATCGGTGGCAATTAAGAATTTCCCCGATCGTTCAAACATTTGGCAATAAAGTTTCTTAAGATTGAATCCTGTTGCCGGTCTTGCGATGATTATCATATAATTTCTGTTGAATTACGTTAAGCATGTAATAATTAACATGTAATGCATGACGTTATTTATGAGATGGGTTTTTATGATTAGAGTCCCGCAATTATACATTTAATACGCGATAGAAAACAAAATATAGCGCGCAAACTAGGATAAATTATCGCGCGCGGTGTCATCTATGTTACTAGATCGG

> fragments of *EV* used in vector construction

GTCCCCAGATTAGCCTTTTCAATTTCAGAAAGAATGCTAACCCACAGATGGTTAGAGAGGCTTACGCAGCAGGTCTCATCAAGACGATCTACCCGAGCAATAATCTCCAGGAAATCAAATACCTTCCCAAGAAGGTTAAAGATGCAGTCAAAAGATTCAGGACTAACTGCATCAAGAACACAGAGAAAGATATATTTCTCAAGATCAGAAGTACTATTCCAGTATGGACGATTCAAGGCTTGCTTCACAAACCAAGGCAAGTAATAGAGATTGGAGTCTCTAAAAAGGTAGTTCCCACTGAATCAAAGGCCATGGAGTCAAAGATTCAAATAGAGGACCTAACAGAACTCGCCGTAAAGACTGGCGAACAGTTCATACAGAGTCTCTTACGACTCAATGACAAGAAGAAAATCTTCGTCAACATGGTGGAGCACGACACACTTGTCTACTCCAAAAATATCAAAGATACAGTCTCAGAAGACCAAAGGGCAATTGAGACTTTTCAACAAAGGGTAATATCCGGAAACCTCCTCGGATTCCATTGCCCAGCTATCTGTCACTTTATTGTGAAGATAGTGGAAAAGGAAGGTGGCTCCTACAAATGCCATCATTGCGATAAAGGAAAGGCCATCGTTGAAGATGCCTCTGCCGACAGTGGTCCCAAAGATGGACCCCCACCCACGAGGAGCATCGTGGAAAAAGAAGACGTTCCAACCACGTCTTCAAAGCAAGTGGATTGATGTGATATCTCCACTGACGTAAGGGATGACGCACAATCCCACTATCCTTCGCAAGACCCTTCCTCTATATAAGGAAGTTCATTTCATTTGGAGAGAACACGGGGGACTCTAGAGGATCTCGAGCGGGGTACCATGGGTAAGGGAGAAGAACTTTTCACTGGAGTTGTCCCAATTCTTGTTGAATTAGATGGTGATGTTAATGGGCACAAATTTTCTGTCAGTGGAGAGGGTGAAGGTGATGCAACATACGGAAAACTTACCCTTAAATTTATTTGCACTACTGGAAAGCTTCCTGTTCCTTGGCCAACACTTGTCACTACTCTTACTTATGGTGTTCAATGCTTTTCAAGATACCCAGATCATATGAAGCGGCACGACTTCTTCAAGAGCGCCATGCCTGAGGGATACGTGCAGGAAAGGACCATCTTCTTCAAGGACGACGGGAACTACAAGACACGTGCTGAAGTCAAGTTTGAGGGAGACACCCTTGTCAACAGGATCGAGCTTAAGGGAATCGATTTCAAGGAGGACGGAAACATCCTCGGCCACAAGTTGGAATACAACTACAACTCCCACAACGTATACATCATGGCAGACAAACAAAAGAATGGAATCAAAGTTAACTTCAAAATTAGACACAACATTGAAGATGGAAGCGTTCAACTAGCAGACCATTATCAACAAAATACTCCAATTGGCGATGGCCCTGTCCTTTTACCAGACAACCATTACCTGTCCACACAATCTGCCCTTTCGAAAGATCCCAACGAAAAGAGAGACCACATGGTCCTTCTTGAGTTTGTAACAGCTGCTGGGATTACACATGGCATGGATGAACTATACAAATAAGAGCTCGAATTTCCCCGATCGTTCAAACATTTGGCAATAAAGTTTCTTAAGATTGAATCCTGTTGCCGGTCTTGCGATGATTATCATATAATTTCTGTTGAATTACGTTAAGCATGTAATAATTAACATGTAATGCATGACGTTATTTATGAGATGGGTTTTTATGATTAGAGTCCCGCAATTATACATTTAATACGCGATAGAAAACAAAATATAGCGCGCAAACTAGGATAAATTATCGCGCGCGGTGTCATCTATGTTACTAGATCGG

> fragments of *Xa7_pro_::Xa7* used in vector construction

CTCAGTTCTACCGGGGTTCAGTTCGGCTGCGGCCTGCAGTGGCTGGCAAAAATCTCGAACTGCTCTGCTCAAGTGCCTCAACTGGCAGAGATAAATCCTAAAAAAACTGAAAAATAGGCCGGTATCCCAGCTCTTCTGCTGAAAAAAACTGAAAAACTGGGTGTAAGATTGATCGCAAAAGTACTTCTGGCACTTGTCATTTTCGCCACTTCTTTGGTCTCTTCGTCCTATTTTTGACATTTCTCTTCGTCCTTTTTTCTTTTCTCTTTCAAACGTGCAGTCGCCGTCGAGGACGGTGAAAGCCCTGACTGCTAAAACCAATATATAACCCCCCCCCCCCCAGATAACCACATACGAACGAAGGCTTTGAAGCATCGCACACTTGAAGAGCCCCCTTCCCAACCACAGCCAGCGGTTTCCAAACTCCACGCCTCGCTCAACCTGGGGGATCCATCATCCATGGCGGCCGCTGATCATCCTGATCGTATGCCCGTTGCAGTTGCAGGCTTGCGCCACCATTACGCCTTCCCTGCAAACCTTCGCCCCGCCGCTCGACTGCTGACCGTCAACTCCGGCGTCTTCCTCATCTCCACCGCCGGGGCCATCGTCCTCGTCCACACCGCCGGTAACCCACCCGCCATCGACAACGATCCAGCCTACGCCTTGGTCGCATTCGTGCTCTTCCTCCTCGGAATCTGGCTCATGTCTATTGCCCTCGTCGCCGACCAGTTCCCGCGCGCCGCTGGGGTCGCCGTGGCCATTGCCAGGGCGCTGCAGGATTACCTCATCGGTGGCAATTAACTAGAAGCTTCGACCATGGCTCTGCACATTCCTCTGCTCCAGTTGTTCCCGGCTTCCCGTACGTGTGCCTGATGATTGTCTTTCTCTGTTTATTTGGCTAGTATTTTAGGCTTGGAAGTTGAAAAACTGTAAATCTGCTTCTTTTTCCCCTCTGTACTACTACTAGACTTTCTTTTTTAAGCTGACGTCATACACACACCCCAGATTCATAACGTGTCGTATAGTAAATGTATTCGAGGCTTGT

> fragments of *Xa7_pro_::Xa10* used in vector construction

CTCAGTTCTACCGGGGTTCAGTTCGGCTGCGGCCTGCAGTGGCTGGCAAAAATCTCGAACTGCTCTGCTCAAGTGCCTCAACTGGCAGAGATAAATCCTAAAAAAACTGAAAAATAGGCCGGTATCCCAGCTCTTCTGCTGAAAAAAACTGAAAAACTGGGTGTAAGATTGATCGCAAAAGTACTTCTGGCACTTGTCATTTTCGCCACTTCTTTGGTCTCTTCGTCCTATTTTTGACATTTCTCTTCGTCCTTTTTTCTTTTCTCTTTCAAACGTGCAGTCGCCGTCGAGGACGGTGAAAGCCCTGACTGCTAAAACCAATATATAACCCCCCCCCCCCCAGATAACCACATACGAACGAAGGCTTTGAAGCATCGCACACTTGAAGAGCCCCCTTCCCAACCACAGCCAGCGGTTTCCAAACTCCACGCCTCGCTCAACCTGGGGGATCCATCATCCATGCAGCTGATGCTCACATTCTGCACGGGCCCCCTCCTGTTTGCCGTCCTCCTACTGATGGTATACCTCAAGCAACTCGCCGCCGCGGCCTGCGTCGACGTGCTCATCATCTACCTCTGCCGCTTCCTCCTCCTCCGCGGCATCATCTTCTCCGGCGACGGCAAGCTACGATTCCGCGTCAAGGTAGCGATCGGGTTCCTCTACATCTCCCTCTCGGCCATACTCTTCTACCTCTCTGCCGCTGTCATGGCGTTGCCGCCGTGGGGTGCGGTGGCCATGTGGGGAATGGCGCTCGTCGCCACTGAGCTTGGCTACTCCTTCTTATGCCCGTATAGCTGCCGCTGCATTGGTGAAGACGACGAGGAGATTTCCCCCGTCTGAGGCCCATATATATCACGATGGATAAACATATTACATACTCCCTCCGTTTCAAAATGTTTGACACCATTGACTTTTCAGCACATGTTTGACCGTTCGTTTCATTCAAAAAAAATTGTGAAATATGTAAAACTATATGTGTACATGGAAATATATTTAACAATGAATCAAATGATATGAAAAGAATAAATAATTACTTAAATTTTTTGAATAAGACGAATGGTGAAACACGTACTAAAAAGTCAATGGTGTCAAACATTTTGAAACGGAGGGAGTATTAATTGGTTTGTTAGTTTGTGTTCATTCATATATAGCTGTTGTATTTTTACGGTTAATAAAGAGAAACCGGCGAGCGCCTAGCAGCCGGCTAGTTTAGTCAAA

> fragments of *Xa7_pro_::Xa23* used in vector construction

CTCAGTTCTACCGGGGTTCAGTTCGGCTGCGGCCTGCAGTGGCTGGCAAAAATCTCGAACTGCTCTGCTCAAGTGCCTCAACTGGCAGAGATAAATCCTAAAAAAACTGAAAAATAGGCCGGTATCCCAGCTCTTCTGCTGAAAAAAACTGAAAAACTGGGTGTAAGATTGATCGCAAAAGTACTTCTGGCACTTGTCATTTTCGCCACTTCTTTGGTCTCTTCGTCCTATTTTTGACATTTCTCTTCGTCCTTTTTTCTTTTCTCTTTCAAACGTGCAGTCGCCGTCGAGGACGGTGAAAGCCCTGACTGCTAAAACCAATATATAACCCCCCCCCCCCCAGATAACCACATACGAACGAAGGCTTTGAAGCATCGCACACTTGAAGAGCCCCCTTCCCAACCACAGCCAGCGGTTTCCAAACTCCACGCCTCGCTCAACCTGGGGGATCCATCATCCATGTTGCATCATCTCAAGGAGCTGGCAGCCGTAGCCGGTATACACATGATCCTCATCTACCTCTGCCGCTTTCTCCTCCGCCGCAGCCGCAACGTATTATTCACCGTTTCCAACAGCCTCCGTTTTCGCCTCAAGGTATTAACTGTATTGTTGTACATATGTCTCTCGGTCATGCTGTTCTACCTGTTTGGCTCCATCATGCCGCTGCCGCCGTGGGGCCTCGTGGTCGGTTGGGTCATGGCCCTCATCGCCGTCGAGCTCGCCTACGCCTTCATCTTTCCATATAGCTTTCGCTACATCGCTGACAACGACGACGACAAGATGGTTATTCTCCCTGTTTAAGCCTTCAGGGCCTATATATATAGTATATATATAAAGCCTTCCATACTGTCTCTTCAATAAAGGCTAGCTTGTGTTGTGAGTTGTATCTGTGTACGTATTTTGTTTGGTTGGTTATATATTGTCACGTAGGTATGCCATATATATATGTATTGCTGTATTTATATTTGTTACTATCTTTTGTTTTTCAGATAATAAAATTCAGCCAGCTTTGCTTGCTTCGTCGTACGTGTATGCTCATCATATCCTCATCCATCAGCTGCTCATAGCTAGCTGGGCCGTGTTATATGTGTGTAGTGATCAGTCACATCCATGTATTTCATCCCATGTATGTTAGTTTGTTTTTCATTTTTGAAAGAAAACATGCATATATGTTATAACTCAAAGTTTTGAGATGAATTTGATCATCTAATTTATTTCCGTTTTGAATTATTCTGCTCTGTTGTTGCAAACTTTTTTTTTTATGTTGGGACACTTTCTCCTCGGTTTGATTGTTATATTGAATTCGTCGATTCCAGCTAGTATATATTTGCTAGTTTCACTCAGACAATCATGCCTATTTGCTGATCAGGAACAATTGAAAACAACCATTACAGAGCCAGACGAAATTAATTAATTTACACTCACCAATTATTCATCATCACATCTCTAAACATCGAATTCTGGGATTCCATCGTTCGTCCCCTTCCCGCAGCCGTCGGATCGCGGATCGACGGTGGCAGATCGCTTCGTTGAACGTTTTTGTAATATACCATTTCTTGAGGGGGGTTTATGCAAAATATCTCTATCCCTTACCTCCTCCTACCTCGCCGGCGTGACGCTCTCCTCCCTCACT

> fragments of *Xa7_pro_::Xa27* used in vector construction

CTCAGTTCTACCGGGGTTCAGTTCGGCTGCGGCCTGCAGTGGCTGGCAAAAATCTCGAACTGCTCTGCTCAAGTGCCTCAACTGGCAGAGATAAATCCTAAAAAAACTGAAAAATAGGCCGGTATCCCAGCTCTTCTGCTGAAAAAAACTGAAAAACTGGGTGTAAGATTGATCGCAAAAGTACTTCTGGCACTTGTCATTTTCGCCACTTCTTTGGTCTCTTCGTCCTATTTTTGACATTTCTCTTCGTCCTTTTTTCTTTTCTCTTTCAAACGTGCAGTCGCCGTCGAGGACGGTGAAAGCCCTGACTGCTAAAACCAATATATAACCCCCCCCCCCCCAGATAACCACATACGAACGAAGGCTTTGAAGCATCGCACACTTGAAGAGCCCCCTTCCCAACCACAGCCAGCGGTTTCCAAACTCCACGCCTCGCTCAACCTGGGGGATCCATCATCCATGGCGGATTGGGCGATGCACCACTACCTCCTACTAGCCAACCAGCAACGCCACCGAGCCCTCGCCGACGTCGCCGTCCGCCGCCGCCAGCTGCTCCTCGACTCCGGCCGCGTCTTCATGCTCCTCGGCGCCGTCATCCTCATGCACATGCTCACCACTACCGGCGGCGGAGCATCGTCCGGCTGCACCCGCGGCGCCGAACCTTGCGTCGCCCTCCTCCTGTGGCTGCTCGGCGCGGCGCTCGCCATGCTGTCGCTCGTCGCCGGCCGATTCCCCGTTCTCGCTGCCGCCATTGCTGAGGAGCTCGGTGATCACCTGCTTGGTGGTCTCTGGTCTCTCTAGTTCTCCTCCGTGTCCGGTGGTCATCTTCTTCTCCGTGCTTTTGCTCTGGAGTTGAGTACGGATCTGTGTGTACTGCATTCTTGCTTAATTAGTGCCCTACACGTTATGCTTTCGAAACATCATCTTTTTTCAGTATAGTTCAATAAATTTCAGCTCAAATTTGTCCTCCAAGACGAGTTCTCCATCCAAACGAAACTTATGGTGTTCCGTTGTTTGGGCCGATTTTATATGTTGGAAATGTACAGACTTCATAG

> fragments of the promoter of *Xa7* used in vector construction

CTCAGTTCTACCGGGGTTCAGTTCGGCTGCGGCCTGCAGTGGCTGGCAAAAATCTCGAACTGCTCTGCTCAAGTGCCTCAACTGGCAGAGATAAATCCTAAAAAAACTGAAAAATAGGCCGGTATCCCAGCTCTTCTGCTGAAAAAAACTGAAAAACTGGGTGTAAGATTGATCGCAAAAGTACTTCTGGCACTTGTCATTTTCGCCACTTCTTTGGTCTCTTCGTCCTATTTTTGACATTTCTCTTCGTCCTTTTTTCTTTTCTCTTTCAAACGTGCAGTCGCCGTCGAGGACGGTGAAAGCCCTGACTGCTAAAACCAATATATAACCCCCCCCCCCCCAGATAACCACATACGAACGAAGGCTTTGAAGCATCGCACACTTGAAGAGCCCCCTTCCCAACCACAGCCAGCGGTTTCCAAACTCCACGCCTCGCTCAACCTGGGGGATCCATCATCC

> fragments of the mutant promoter of *Xa7* used in vector construction

CTCAGTTCTACCGGGGTTCAGTTCGGCTGCGGCCTGCAGTGGCTGGCAAAAATCTCGAACTGCTCTGCTCAAGTGCCTCAACTGGCAGAGATAAATCCTAAAAAAACTGAAAAATAGGCCGGTATCCCAGCTCTTCTGCTGAAAAAAACTGAAAAACTGGGTGTAAGATTGATCGCAAAAGTACTTCTGGCACTTGTCATTTTCGCCACTTCTTTGGTCTCTTCGTCCTATTTTTGACATTTCTCTTCGTCCTTTTTTCTTTTCTCTTTCAAACGTGCAGTCGCCGTCGAGGACGGTGAAAGCCCTGACTGCTAAAACCAATATATAACCCCCCCCCCCAGATAACCACATACGAACGAAGGCTTTGAAGCATCGCACACTTGAAGAGCCCCCTTCCCAACCACAGCCAGCGGTTTCCAAACTCCACGCCTCGCTCAACCTGGGGGATCCATCATCC
